# Insanity and Its Treatment

**Published:** 1886-07

**Authors:** 


### Book Reviews.

Insanity and its Treatment. *Lectures on the Treatment, Medical and Legal, of Insane Patients. By* G. Fielding Blandford, m. d., *Oxon; Fellow of the Royal College of Physicians in London; late Lecturer on Psychological Medicine at the School of St. George's Hospital, London. Third Edition. Together with* Types of Insanity, *an Illustrated Guide in the Physical Diagnosis of Mental Disease, by* Allan McLane Hamilton, m. d., *one of the Consulting Physicians to the Insane Asylums of New York City, and the Hudson River State Hospital for the Insane, etc. Octavo, pp. ix, 379. New York:* William Wood & Company. *Chicago:* W. T. Keener.

That this work has lived to see its third edition is evidence of its popularity and its usefulness. It is for the most part re-written, and the subject is brought down to the present time. It is not, nor is the claim made, that it is a systematic treatise, but is rather a hand-book for the use of students and practitioners—a guide for those who have to deal with insanity as it comes to them in general practice. For these it is perhaps the best work in our language; at the same time we think there are few English-speaking specialists who do not possess a copy of this work.

The appendix, on Types of Insanity, by Dr. Hamilton, adds materially to the value of the book. The nine plates illustrating the different forms of insanity are interesting and typical.

Their value in the diagnosis of mental disorders is very doubtful, as has been shown in the asylums of Venice, where the physiognomy of mental disease has been fully investigated.

